# Sick Leave and Work Participation Among Parents of Children with Autism Spectrum Disorder in the Stockholm Youth Cohort: A Register Linkage Study in Stockholm, Sweden

**DOI:** 10.1007/s10803-015-2381-1

**Published:** 2015-02-20

**Authors:** Miranda McEvilly, Susanne Wicks, Christina Dalman

**Affiliations:** 1 Department of Public Health Sciences, Karolinska Institutet, Stockholm, Sweden; 2 Sorögatan 29, 16447 Kista, Sweden

**Keywords:** Autism spectrum disorder (ASD), Sick leave, Epidemiology, Register study

## Abstract

This population-based register study explored the association between having a child with/without autism spectrum disorder (ASD) and parental sick leave and work participation. Parents of children with ASD living in Stockholm, Sweden in 2006 were more likely to be on sick leave, not in the labor force, or earning low income when compared to parents who did not have a child with ASD and these results remained after adjusting for familial socioeconomic factors and parental psychiatric care. Sick leave among parents was associated with having a child with ASD without intellectual disability (ID) but not ASD with ID. Although Sweden has policies helping families with children with ASD this study suggests that there exist unmet needs among these parents.

## Introduction

In the past decades the prevalence of diagnosed autism spectrum disorder (ASD) has been increasing in the world (Fombonne 2009), and in 2007 an estimated 1 % of children had ASD in Stockholm County, Sweden (Idring et al. 2012). In the United States of America, parental reported ASD prevalence rose from 1.2 % in 2007 to 2.0 % in 2011–2012 (Blumberg et al. 2013) with much of the increase coming from children who have ASD without intellectual disabilities (ID).Although some of the increase can be explained by a widening of diagnostic criteria, other contributing factors include public awareness and societal demand (Fombonne 2012). The increase in ASD prevalence impacts not only children with ASD but their families as well. Several studies have associated parenting a child with ASD with adverse health outcomes when compared to parents of typically developing (TD) children. Adverse health outcomes include high levels of stress (Hayes and Watson 2013) depression (Van Steijn 2013), fatigue (Giallo et al. 2011), poor sleep (Meltzer 2008), and self-rated poor health (Allik et al. 2006). However, few studies have examined sick leave and work participation among parents who have a child with ASD and even fewer such studies have been performed in Sweden. Sweden has one of the world’s most developed support systems in place, including laws and compensatory measures, to enable all parents to work, with additional support and services available to families who have a child with a disability. Thus, it is of interest to examine sick leave and work participation in this setting. Hitherto, to our knowledge, there have been no studies on sick leave and only two studies touching upon the subject of work participation. Olsson and Hwang, studying families of children with ID (regardless of other diagnosis) found that the mother’s but not the father’s work participation was affected by having a child with an ID (Olsson and Hwang 2003). However, their study only included 68 children with autism (total number of children with ID was 226) and did not include children with autism without ID. The second study examined the costs of having a child with ASD and indicated that parents lose income when abstaining from paid work (Järbrink 2007). Järbrink et al’s study also had a limited sample size (n = 33) and the subjects were recruited from the local child health center rather than the total population.

Sweden has many policies to promote combining career with family. Parental insurance allows parents to stay home after the birth of a child for 480 days (The Swedish Social Insurance Agency 2009), but after this period parents usually return to work. Most children in Sweden attend daycare (83 % of all two year olds in 2006) (Swedish National Agency for Education 2013) and a high percentage of parents are classified as being in the work force (82 % mothers and 92 % fathers) (Statistics Sweden 2013) with 42 % of mothers and 74 % of fathers working full time (Statistics Sweden 2013). Parents may stay home to care for sick children, they are entitled to work part time, and daycare is heavily subsidized. Sweden has an individual payer tax system which provides an incentive for both parents to work and there are laws requiring employers to help “both female and male employees to combine employment and parenthood” (Discrimination Act (SFS 2008:567)). Parents who have children with disabilities are given additional help in order to combine work with parenthood; for example they may take off ten extra days per year with compensation to attend parental education or health appointments. All parents in Sweden are given a monthly child allowance per child and parents with a child with a disability may be eligible for and can apply for an extra financial support, care allowance (Swedish Social Insurance Agency 2009).

Sick leave benefits in Sweden are paid out from the second day of illness, with the first fourteen days being paid for by the employer and days extending beyond two weeks paid for by the Swedish National Social Insurance Agency. A doctor’s certificate is required to retain sick leave benefits after the first week. (Swedish National Insurance Act (1962:381) and Swedish Sick Pay Act (1991:1074).

This comprehensive register-based study compares sick leave and work participation among parents with a child with ASD to that of parents who do not have a child with ASD (but who may have a child with another disability) in Stockholm, Sweden in 2006. The study examines a large number of parents with children with ASD (with or without ID) using objective data from local and National statistics. The aim of the study is to examine whether or not the parents differ in the amount of sick leave they take and in their work participation. A secondary aim is to differentiate between parents having a child with ASD with or without ID.

## Methods

### Study Population, Participants

The study population consisted of the biological families (n = 326,183) of children in the Stockholm Youth Cohort (SYC) previously described by Idring et al., a cohort including all children ages 0–17 who resided in Stockholm County at some time in the years 2001–2007 (Idring et al. 2012). The following exclusion criteria were applied to the study population: Families without any children aged 4–17 in 2006 (n = 116,986) were excluded because children under four years old are unlikely to have been diagnosed with ASD. Families where the 4–17 year old/s had not lived in Stockholm from 2001–2006 (2002–2006 for four year olds) (n = 27,705) were excluded to ensure they had adequate time be diagnosed/registered in the Stockholm health care system. Families in which the mother gave birth in 2006 or 2007 (n = 9,774) were excluded since approximately 21 % of women who were pregnant in 2006 and 2007 received pregnancy benefits, which are presented together with sickness benefits data in the sources from the Swedish Social Insurance. Families with more than six children (n = 766) were excluded because having a large family may in itself impact parental sick leave and work participation. Parents who had children with more than one partner potentially could have been represented multiple times and in order to prevent this, only the family from the first partner was kept and subsequent families were excluded (n = 11,586). Families who had a child with ASD, but that child was not in the 4–17 age range were excluded (n = 413). Families with missing data were excluded, both those who were absent from the longitudinal integration database for health insurance and labor market studies (LISA), (Statistics Sweden 2011a) (n = 4,350) and those who were present in LISA but were missing data on sick leave and/or work participation (n = 5,036). After all exclusions, the final analytical sample consisted of 149,567 families.

### Exposure Assessment

Biological families (mother, father and siblings) were identified via the Multigenerational Register (Statistics Sweden 2011b) using record linkage. Children with ASD were identified by Idring et al. with a multiple (four) register approach in order to maximize case identification, and covering ASD case status as of December 31, 2007. One register used was the Clinical Database for Child and Adolescent Psychiatry in Stockholm which covers in and outpatient care within Stockholm since 2001 as well as diagnoses based on DSM IV (American Psychiatric Association 1994). A second source was the outpatient care from the VAL databases, which covers public health care services since 1997 and includes diagnoses from ICD (World Health Organization (WHO) 1992) with good coverage of diagnosis from 2006. A third source was the Habilitation Register, which records the use of habilitation services in Stockholm since 1998. The Habilitation services center helps individuals with disabilities, including but not limited to ASD, and having a diagnosis is a prerequisite for receiving services. Finally, the inpatient care from the National patient register, with as good as complete coverage for psychiatric clinics from 1973 onwards and which includes diagnoses from the ICD systems, was used. The first three of these four registers are maintained by the Stockholm County Council (SLL), while the fourth register is maintained by the National Board of Health and Welfare (Ludvigsson et al. 2011). Additionally, Idring et al. identified ASD with and without ID using recorded diagnoses meeting criteria for intellectual disability in ICD 9 (317–319), ICD 10 (F70–79) and DSM IV (317–319) and using records from the Habilitation register which categorize recipients into having autism with or without ID. These ASD cases were validated by Idring et al. (2012) in a study where they examined 177 randomly selected cases. Of these 96 % were accurate with an ASD diagnosis according to the journals. Idring et al. also cross validated SYC cases with a twin study including questionnaire data (Lichtenstein et al. 2010).

### Measures

#### Outcomes

Four outcomes, two for sick leave and two for work participation, were obtained using data from LISA in 2006. The first outcome for sick leave (sick for 15–365 days) measured if a person had received or not received sickness benefits from the Swedish Social Insurance, which are paid out after two weeks and up to one year. The second outcome for sick leave measured if a person had been deemed unable to work for longer than one year and were therefore receiving activity or sickness compensation benefits (long term sick leave/deemed unable to work). To examine work participation we looked at being in the labor force (being employed) and at parental income. Being employed is measured by Statistics Sweden in the month of November, a month with less variability in the work force compared to summer and months with holidays. The income studied was income from employment (not income from other sources such as government assistance). Having a very low income (below the 20 % percentile) was considered a crude measure of increased likelihood of part time employment.

#### Exposure

Parents having a child aged 4–17 with ASD in 2006 were classified as exposed. This exposure was further stratified by whether the child had ASD with or without ID. Families having more than one child with ASD where both with and without ID were represented (n = 25) were classified as having a child with ASD with ID.

#### Covariates

Covariates were selected based on the likelihood that they might be associated with the outcomes and the exposures, and therefore needed to be considered as possible confounders. Parents were classified as having/not having psychiatric contact prior to becoming a parent. Psychiatric contact was defined in one of two ways: first, being in inpatient care at a psychiatric clinic or a clinic for those with an addiction problem in the National Patient Register, Sweden, or having a psychiatric diagnosis (from any health entity) from 1973 onwards and second, visiting a psychiatric or addiction clinic in Stockholm (VAL) in the years 1997 onwards. In addition to the covariates parental age (< 35, 35–44 and > 44 years), parental birth country (born inside or outside of Sweden) and family size (1, 2, or 3 or more children between the ages 4–17 years), we also looked at the following socioeconomic covariates: single parent/two parent households (dichotomy), parental education (9 years or less, 10–12 years, more than 12 years, unknown) and social assistance (parent receiving social assistance during 2006 or not).

### Data Analysis

Multiple logistic regressions were performed to obtain odds ratios (OR) with confidence intervals (CI) of 95 % to examine the association between sick leave, long term sick leave, not being in the labor force, low income and having a child with or without ASD. In the first model we adjusted for parental age. In the second model we introduced parental contact with psychiatric care prior to birth of first child. In the third model we added the potentially confounding covariates parental birth country and number of children aged 4–17 in the family. In the final model we adjusted for socioeconomic covariates; parental education, two parent/single parent household, and receiving social assistance. Being a parent to a child with ASD was also analyzed stratified by ASD with or without ID. After the final model, we also checked the effect of mother’s/father’s situation by adjusting for the other parent’s covariates and each outcome. SAS 9.2 (SAS Institute Inc., Cary, NC, USA) was used to perform statistical analysis.

Ethical permission for this study was obtained from the Regional Ethical Review Board in Stockholm (2007/545-31).

## Results

Our final sample included 149,567 mothers and 149,567 fathers, all having a child 4–17 years old in 2006. The number of mothers/fathers who had a 4–17 year old child with ASD was 2,892, and of these 1,207 had ASD with ID (or more than one child with ASD where at least one of the children had ASD with ID) and 1,685 had ASD without ID.

### Descriptive Results

Descriptive results are shown in Tables 1 and 2. The proportion of parents with a child with ASD taking sick leave and not being in the labor force or earning less was higher than for parents without a child with ASD. A higher percentage of parents who took sick leave, were not in the labor force or had a low income were born outside of Sweden. For example 10.5 % of fathers born outside of Sweden were on long term sick leave versus 2.7 % of fathers born in Sweden, and 29.6 % of mothers born outside of Sweden were not in the labor force versus 10.0 % of mothers born in Sweden. Likewise, parents with less than 10 years of education, single household parents, or parents receiving social assistance were more likely to have all four outcomes. For example, 36 % of mothers with less than 10 years of education were not in the labor force versus 9.7 % of mothers who have more than 12 years education, and 44.4 % of lone fathers earned a low income versus 27.5 % of fathers living in a two parent household. Parental contact with psychiatric care prior to the birth the first child impacted the outcomes with a higher proportion of these parents being on sick leave, not being in the labor force, and earning a low income. For example 17.2 % of fathers and 14.2 % of mothers among this group were on long term sick leave.

**Table 1:** Descriptive statistics of prevalence of exposure variables and other covariates among parents (n = 149,567) taking sick leave and long term sick leave

Variables	Mother sick leave >14 days		Father sick leave >14 days		Mother long term sick leave		Father long term sick leave	
	No	Yes	No	Yes	No	Yes	No	Yes
	% (n)	% (n)	% (n)	% (n)	% (n)	% (n)	% (n)	% (n)
*Exposure*								
No ASD	85.0 (124,723)	15.0 (21,952)	91.2 (133,737)	8.8 (12,938)	94.0 (137,853)	6.0 (8,822)	95.6 (140,151)	4.5 (6,524)
ASD	81.4 (2,354)	18.6 (538)	89.5 (2,589)	10.5 (303)	88.5 (2,558)	11.6 (334)	93.5 (2,703)	6.5 (189)
ASD (without ID)	79.8 (1,345)	20.2 (340)	89.1 (1,502)	10.9 (183)	88.0 (1,482)	12.0 (203)	94.4 (1,590)	5.6 (95)
ASD (with ID)	83.6 (1,009)	16.4 (198)	90.1 (1,087)	9.9 (120)	89.2 (1,076)	10.9 (131)	92.2 (1,113)	7.8 (94)
*Descriptive*								
Parental age								
< 35	84.0 (17,531)	16.0 (3,344)	91.5 (11,037)	8.5 (1,024)	97.2 (20,298)	2.8 (577)	98.5 (11,875)	1.5 (186)
35–44	85.3 (69,520)	14.7 (11,944)	91.7 (63,318)	8.1 (5,609)	94.9 (77,321)	5.1 (4,143)	97.5 (67,171)	2.6 (1,756)
> 44	84.8 (40,026)	15.3 (7,202)	90.4 (61,971)	9.6 (6,608)	90.6 (42,792)	9.4 (4,436)	93.0 (63,808)	7.0 (4,771)
Birth country								
Sweden	85.3 (98,523)	14.7 (16,996)	92.1 (105,643)	7.9 (9,102)	95.0 (109,725)	5.0 (5,794)	97.3 (111,681)	2.7 (3,064)
Not Sweden	83.9 (28,554)	16.1 (5,494)	88.1 (30,683)	11.9 (4,139)	90.1 (30,686)	9.9 (3,362)	89.5 (31,173)	10.5 (3,649)
Children 4–17 years								
1	83.8 (60,619)	16.2 (11,723)	90.5 (65,499)	9.5 (6,843)	92.4 (66,864)	7.6 (5,478)	94.6 (68,439)	5.4 (3,903)
2	86.0 (53,822)	13.9 (8,745)	91.8 (57,431)	8.2 (5,136)	95.3 (59,613)	4.7 (2,954)	96.6 (60,435)	3.4 (2,132)
3 or more	86.2 (12,636)	13.8 (2,022)	91.4 (13,396)	8.6 (1,262)	95.1 (13,934)	4.9 (724)	95.4 (13,980)	4.6 (678)
*Socioeconomic*								
Education*								
≤ 9 years	81.1 (12,884)	18.9 (3,003)	87.7 (19,111)	12.3 (2,686)	85.0 (13,500)	15.0 (2,387)	90.4 (19,693)	9.7 (2,104)
10–12 years	82.7 (55,111)	17.3 (11,502)	89.6 (59,152)	10.4 (6,874)	93.1 (61,990)	6.9 (4,623)	95.3 (62,894)	4.7 (3,132)
> 12 years	88.1 (58,599)	11.9 (7,947)	94.1 (57,387)	6.0 (3,630)	96.9 (64,457)	3.1 (2,089)	97.7 (59,633)	2.3 (1,384)
2 parent household*								
Yes	86.5 (94,310)	13.5 (14,680)	92.0 (100,251)	8.0 (8,739)	95.2 (103,699)	4.9 (5,291)	96.5 (105,221)	3.5 (3,769)
No	80.7 (32,438)	19.3 (7,758)	88.9 (35,728)	11.1 (4,468)	90.7 (36,454)	9.3 (3,742)	92.9 (37,344)	7.1 (2,852)
Social assistance								
No	85.0 (121,927)	15.0 (21,446)	91.3 (131,450)	8.8 (12,605)	94.3 (135,223)	5.7 (8,150)	96.0 (138,299)	4.0 (5,756)
Yes	83.1 (5,150)	16.9 (1,044)	88.5 (4,876)	11.5 (636)	83.8 (5,188)	16.2 (1,006)	82.6 (4,555)	17.4 (957)
*Other*								
Parental psychiatric contact prior to parenthood								
Yes	89.4 (20,102)	10.62 (2,388)	89.7 (11,883)	10.3 (1,358)	85.8 (5,561)	14.2 (1,299)	82.8 (5,561)	17.2 (1,152)
No	92.6 (118,122)	7.1 (8,955)	92.7 (126,341)	7.3 (9,985)	92.9 (130,367)	7.2 (10,044)	92.9 (132,663)	7.1 (10,191)

** Missing variables: education: mothers 0.35 (n = 521) and fathers 0.49 (n = 727), household 0.25 (n = 381)

**Table 2:** Descriptive statistics of prevalence of exposure variables and other covariates among not in the labor force and low income mothers and fathers (n = 149,567)

Variables	Mother		Father		Mother low income		Father low income	
	In labor force	Not in labor force	In labor force	Not in labor force	No	Yes	No	Yes
	% (n)	% (n)	% (n)	% (n)	% (n)	% (n)	% (n)	% (n)
*Exposure*								
No ASD	85.7 (125,666)	14.3 (21,009)	87.3 (128,033)	12.7 (18,642)	70.1 (102,782)	29.9 (43,893)	67.9 (99,638)	32.1 (47,037)
ASD	79.4 (2,297)	20.6 (595)	84.2 (2,436)	15.8 (456)	61.8 (1,788)	38.2 (1,104)	64.1 (1,855)	35.9 (1,037)
ASD (without ID)	80.7 (1,360)	19.3 (325)	85.4 (1,439)	14.6 (246)	63.4 (1,069)	36.6 (616)	65.2 (1,099)	34.8 (586)
ASD (with ID)	77.6 (937)	22.4 (270)	82.6 (997)	17.4 (210)	59.6 (719)	40.4 (488)	62.6 (756)	37.4 (451)
*Descriptive*								
Parental Age								
<35	75.8 (15,819)	24.2 (5,056)	85.1 (10,263)	14.9 (1,798)	50.9 (10,618)	49.1 (10,257)	57.9 (6,985)	42.1 (5,076)
35–44	87.1 (70,980)	12.9 (10,484)	90.2 (62,163)	9.8 (6,764)	71.8 (58,498)	28.2 (22,966)	71.4 (49,237)	28.6 (19,690)
>44	87.2 (41,164)	12.8 (6,064)	84.6 (58,043)	15.4 (10,536)	75.1 (35,454)	24.9 (11,774)	66.0 (45,271)	34.0 (23,308)
Birth country								
Sweden	90.0 (103,985)	10.0 (11,534)	91.6 (105,075)	8.4 (9,670)	75.0 (86,597)	25.0 (28,922)	75.4 (86,498)	24.6 (28,247)
Not Sweden	70.4 (23,978)	29.6 (10,070)	72.9 (25,394)	27.1 (9,428)	52.8 (17,973)	47.2 (16,075)	43.1 (14,995)	56.9 (19,827)
Children 4–17 years								
1	83.9 (60,750)	16.0 (11,592)	84.8 (61,310)	15.3 (11,032)	66.9 (48,418)	33.1 (23,924)	63.7 (46,054)	36.3 (26,288)
2	88.3 (55,268)	11.7 (7,299)	90.1 (56,400)	9.9 (6,167)	74.5 (46,585)	25.5 (15,982)	72.9 (45,592)	27.1 (16,975)
3 or more	81.5 (11,945)	18.5 (2,713)	87.0 (12,759)	13.0 (1,899)	65.3 (9,567)	34.7 (5,091)	67.2 (9,847)	32.8 (4,811)
*Socioeconomic*								
Education*								
≤9 years	64.2 (10,192)	35.9 (5,695)	74.5 (16,247)	25.5 (5,550)	44.3 (7,038)	55.7 (8,849)	45.7 (9,957)	54.3 (11,840)
10–12 years	86.3 (57,476)	13.7 (9,137)	87.4 (57,719)	12.6 (8,307)	68.9 (45,882)	31.1 (20,731)	65.6 (43,277)	34.5 (22,749)
>12 years	90.3 (60,104)	9.7 (6,442)	91.9 (56,100)	8.1 (4,917)	77.4 (51,532)	22.6 (15,014)	78.7 (48,020)	21.3 (12,997)
2 Parent household*								
Yes	87.14 (94,975)	12.9 (14,015)	90.6 (98,748)	9.4 (10,242)	71.3 (77,711)	28.7 (31,279)	72.5 (79,029)	27.5 (29,961)
No	81.7 (32,829)	18.3 (7,367)	78.4 (31,524)	21.6 (8,672)	66.5 (26,747)	33.5 (13,449)	55.6 (22,345)	44.4 (17,851)
Social assistance								
No	87.9 (125,959)	12.2 (17,414)	89.4 (128,797)	10.6 (15,258)	72.26 (103,599)	27.7 (39,774)	70.1 (100,977)	29.9 (43,078)
Yes	32.4 (2,004)	67.7 (4,190)	30.3 (1,672)	69.7 (3,840)	15.7 (971)	84.3 (5,223)	9.4 (516)	90.6 (4,996)
*Other*								
Parental psychiatric contact prior to birth of first child								
Yes	87.0 (18,792)	13.0 (2,812)	86.2 (16,459)	13.8 (2,639)	88.5 (39,824)	11.5 (5,173)	88.9 (42,734)	11.1 (5,340)
No	93.3 (119,432)	6.7 (8,531)	93.3 (121,765)	6.7 (8,704)	94.1 (98,400)	5.9 (6,170)	94.1 (95,490)	5.9 (6,003)

** Missing variables: education: mothers 0.35 (n = 521) and fathers 0.49 (n = 727), household 0.25 (n = 381)

### Analytical Results

Parents of children who have ASD were more likely to take sick leave (measured by sick leave and long term sick leave) and to have lower work participation (measured by not being in the labor force and low income) than other parents (Tables 3, 4). A significant association was seen with all outcomes for mothers and fathers of a child with ASD, with the exception of fathers taking sick leave 15–365 days in model 4. The association was stronger among mothers. For mothers, the OR for sick leave/long term sick leave if they had a child with ASD was OR 1.3 (95 % CI 1.2–1.4)/OR 2.0 (95 % CI 1.8–2.3) in the crude model, while for fathers they were OR 1.2 (95 % CI 1.1–1.4)/OR 1.5 (95 % CI 1.2–1.7). For not being in the labor force and low income the results were OR 1.6 (95 % CI 1.5–1.8) and OR 1.5 (95 % CI 1.4–1.6) for mothers, and OR 1.3 (95 % CI 1.2–1.4) and OR 1.2 (95 % CI 1.1–1.3) for fathers. When stratifying by ASD with or without ID, the association with sick leave 15–365 days was strengthened among mothers and fathers who had a child with ASD without ID and was not significant among mothers and fathers who had a child with ASD with ID. The association with long term sick leave was strengthened for mothers of children with ASD without ID and weakened, but still significant, for mothers of children with ASD with ID. Among fathers of children with ASD with ID the association with long term sick leave was strengthened and the association among fathers of children with ASD without ID was weakened. Having a child with ASD with ID strengthened the association to not being in the labor force for both mothers and fathers. Having a child with ASD without ID weakened the association with not being in the labor force, and among fathers the association was not significant in all models. Finally, when considering low income, the associations were strengthened for mothers and fathers who had a child with ASD with ID and weakened for a child with ASD without ID, but the association remained. The OR was weakened slightly when adjusting for parental contact with psychiatric care. When adjusting for country of birth and the number of children the results were slightly strengthened. In the final model, the associations with outcomes of parents with a child with ASD/ASD without ID/ASD with ID and sick leave tended to be slightly weakened. In the final model for work participation the results were similar for ASD, slightly weakened for ASD with ID and strengthened for ASD without ID. In addition to the four models mentioned above, the data was re-analyzed with adjustments for the other parent’s covariates and outcomes. These extra analyses did not change the results.

**Table 3:** Odds Ratios (ORs) and 95 % Confidence Intervals (CI) of the association of sick leave (15-365 days) and long term sick leave (more than 365 days) with being a parent without a child with Autism Spectrum Disorder (ASD), a parent of a child with ASD (with or without Intellectual Disability (ID)) a parent of a child with ASD without ID, or a parent of a child with ASD with ID

	Mothers				Fathers			
	Model 1 OR (95 % CI)	Model 2 OR (95 % CI)	Model 3 OR (95 % CI)	Model 4 OR (95 % CI)	Model 1 OR (95 % CI)	Model 2 OR (95 % CI)	Model 3 OR (95 % CI)	Model 4 OR (95 % CI)
Sick leave 15–365 days								
No ASD	1	1	1	1	1	1	1	1
ASD	**1.3 (1.2–1.4)**	**1.3 (1.2–1.4)**	**1.3 (1.2–1.5)**	**1.3 (1.2–1.4)**	**1.2 (1.1–1.4)**	**1.2 (1.1–1.3)**	**1.2 (1.1–1.4)**	1.2 (1.0**–**1.3)
ASD (without ID)	**1.4 (1.3–1.6)**	**1.4 (1.3–1.6)**	**1.5 (1.3–1.6)**	**1.4 (1.3–1.6)**	**1.3 (1.1–1.5)**	**1.2 (1.1–1.5)**	**1.3 (1.1–1.5)**	**1.2 (1.1–1.5)**
ASD (with ID)	1.1 (1.0–1.3)	1.1 (1.0**–**1.3)	1.1 (1.0**–**1.3)	1.1 (0.9**–**1.3)	1.1 (0.9**–**1.4)	1.1 (0.9**–**1.4)	1.1 (0.9**–**1.3)	1.1 (0.9**–**1.3)
Long term sick leave								
No ASD	1	1	1	1	1	1	1	1
ASD	**2.0 (1.8–2.3)**	**2.0 (1.7–2.2)**	**2.1 (1.9–2.4)**	**2.0 (1.7–2.2)**	**1.5 (1.2–1.7)**	**1.4 (1.2–1.6)**	**1.5 (1.3–1.7)**	**1.4 (1.1–1.6)**
ASD (without ID)	**2.1 (1.8–2.5)**	**2.1 (1.8–2.4)**	**2.3 (2.0–2.7)**	**2.2 (1.9–2.6)**	**1.3 (1.0–1.5)**	1.2 (1.0**–**1.5)	**1.4 (1.1–1.7)**	1.2 (1.0**–**1.5)
ASD (with ID)	**1.9 (1.6–2.2)**	**1.8 (1.5–2.2)**	**1.9 (1.6–2.3)**	**1.7 (1.4–2.1)**	**1.7 (1.4–2.1)**	**1.6 (1.3–2.0)**	**1.6 (1.3–2.0)**	**1.5 (1.2–1.9)**

Estimates with 95 % Confidence Intervals that do not incorporate 1.00, are shown in bold

*ORs* Odds Ratios; *CI* Confidence Intervals; *ASD* Autism Spectrum Disorder; *ID* Intellectual Disability

Model 1: adjusted for parental age

Model 2: Model 1 + parental (either mother or father) contact with psychiatric care prior to birth of first child

Model 3: Models 1 and 2 + country of birth and number of children

Model 4: Models 1, 2 and 3 + education, two parent/lone parent household and social assistance

**Table 4:** Odds Ratios (ORs) and 95 % Confidence Intervals (CI) of the association of not being in the labor force and low income with being a parent without a child with Autism Spectrum Disorder (ASD), a parent of a child with ASD (with or without Intellectual Disability (ID)) a parent of a child with ASD without ID, or a parent of a child with ASD with ID

	Mothers				Fathers			
	Model 1 OR (95 % CI)	Model 2 OR (95 % CI)	Model 3 OR (95 % CI)	Model 4 OR (95 % CI)	Model 1 OR (95 % CI)	Model 2 OR (95 % CI)	Model 3 OR (95 % CI)	Model 4 OR (95 % CI)
Not in labor force								
No ASD	1	1	1	1	1	1	1	1
ASD	**1.6 (1.5–1.8)**	**1.6 (1.4–1.7)**	**1.6 (1.5–1.8)**	**1.6 (1.4–1.8)**	**1.3 (1.2–1.4)**	**1.3 (1.1–1.4)**	**1.3 (1.2–1.5)**	**1.2 (1.1–1.4)**
ASD (without ID)	**1.5 (1.3–1.7)**	**1.4 (1.3–1.6)**	**1.6 (1.4–1.9)**	**1.6 (1.4–1.8)**	1.2 (1.0**–**1.3)	1.1 (1.0**–**1.3)	**1.3 (1.1–1.5)**	1.2 (1.0**–**1.4)
ASD (with ID)	**1.8 (1.6–2.0)**	**1.8 (1.5–2.0)**	**1.7 (1.4–1.9)**	**1.6 (1.3–1.8)**	**1.4 (1.2–1.7)**	**1.4 (1.2–1.6)**	**1.4 (1.2–1.6)**	**1.3 (1.1–1.5)**
Low income								
No ASD	1	1	1	1	1	1	1	1
ASD	**1.5 (1.4–1.6)**	**1.5 (1.4–1.6)**	**1.6 (1.4–1.7)**	**1.5 (1.4–1.6)**	**1.2 (1.1–1.3)**	**1.2 (1.1–1.3)**	**1.2 (1.2–1.4)**	**1.2 (1.1–1.3)**
ASD (without ID)	**1.4 (1.3–1.6)**	**1.4 (1.2–1.5)**	**1.5 (1.4–1.7)**	**1.5 (1.3–1.7)**	1.1 (1.0**–**1.3)	1.1 (1.0**–**1.2)	**1.3 (1.1–1.4)**	**1.2 (1.1–1.3)**
ASD (with ID)	**1.7 (1.5–1.9)**	**1.6 (1.5–1.8)**	**1.6 (1.4–1.8)**	**1.6 (1.4–1.8)**	**1.3 (1.1–1.4)**	**1.3 (1.1–1.4)**	**1.2 (1.1–1.4)**	1.2 (1.0**–**1.3)

Estimates with 95 % Confidence Intervals that do not incorporate 1.00, are shown in bold

*ORs* Odds Ratios; *CI* Confidence Intervals; *ASD* Autism Spectrum Disorder; *ID* Intellectual Disability

Model 1: adjusted for parental age

Model 2: Model 1 + parental (either mother or father) contact with psychiatric care prior to birth of first child

Model 3: Models 1 and 2 + country of birth and number of children

Model 4: Models 1, 2 and 3 + education, two parent/lone parent household and social assistance

## Discussion

In this population based study, taking possible confounders into account, we found that parents of children with ASD, especially mothers, were on sick leave more often and participated less in work compared to parents of children without ASD. The findings occur despite the fact that Sweden provides extra compensatory measures of support to parents of children with disabilities in order to enable their successful participation in the work force and a healthy life. Differences between parents with and without a child with ASD remain after accounting for educational level, being a lone parent, and receiving social assistance. When stratifying parents with ASD child into ASD with or without ID some differences are found. Increased sick leave (15–365 days) is associated with parents of children with ASD without ID but not ASD with ID. Increased long term sick leave (over one year) is more strongly associated with mothers of children with ASD without ID and, in contrast, fathers to children with ASD with ID. Parents with a child with ASD with ID are more likely to be outside of the labor force or have low income than parents with a child with ASD without ID, but this difference is weaker in the final model.

Parents who have a child with ASD are more likely to experience stress, depression, and fatigue. Therefore it is not surprising that these parents take sick leave more frequently or participate less in the work force. However, it is interesting to see that differences remain in Sweden where there is universal health care and children and families with disabilities are entitled to extra help, help that is meant to allow all families to have similar conditions. It is also interesting to consider why parents with children who have ASD without ID are more likely to be on sick leave (15–365 days) than parents with a child with ASD with ID. A possible explanation might be that these parents experience a higher level of burden. Jones et al. have suggested that parental anxiety might be higher when a child has ASD with a higher level of adaptive functioning since that child is likely to live more independently than a child with ASD with a lower level of functioning, exposing them to dangers (Jones et al. 2013). Parental anxiety might lead to sleep deprivation or missed work. Another explanation is that children with ASD without ID are more likely to suffer from comorbid anxiety and depression (Mayes et al. 2011) than children with ASD with ID, which affects not only the child but the entire family. Finally, another reason could be that families with a child with ASD without ID might not get adequate help. Societal help in Sweden is given based on the individual child’s need but in Olsson and Hwang’s Swedish study on children with three different types of ID (Down’s syndrome, ASD with ID, and other ID) they found that more familial financial support (care allowance) was given to children with DS even though they had less needs than the children with ID or ASD with ID (Olsson and Hwang 2003); it might be more difficult to get help for a child who has a disability that is not visible or a disability which is varied (for example a child who is gifted in some areas and behind in other areas).

### Strengths and Limitations

The main strength of the study is that it was based on a cohort containing the total population of children 0–17 years living in Stockholm sometime between 2001 and 2007 and that Sweden has high quality registers (Allebeck 2009) enabling us to identify parents for the study using record linkage. Additional strengths are that Sweden has universal health care which increases the likelihood that ASD cases are detected (Idring et al. 2012), and that the ASD cases in the study are validated (see “Methods”). Finally, because the study looks at sick leave that exceeds two weeks it is unlikely that sick leave goes unreported. People with flexible jobs might not report when they are sick if they can make up missed work on weekends or other times, but this would be hard to maintain for sickness lasting two weeks. Also, in order to be on sick leave a doctor’s certificate is required and a person on sick leave must continually return to the doctor to get their sick leave extended. Once deemed fit to work the person is required to return to or look for work, which means that parents in the study are not “choosing” to be on sick leave. Additionally, people on social benefits are required to do activities to help them reenter the work force which makes it less likely that parents “choose” to be receiving social benefits.

Despite the strengths, there are several limitations with our study which should be noted. Although we controlled for parental psychiatric care before birth of first child we believe residual confounding might occur if parents have psychiatric problems and have not sought psychiatric care. Psychiatric illness proceeding parenthood results in the problem of reverse causality preventing us from distinguishing between the parent’s condition and the effect of parenting a child with ASD. Another limitation is that despite universal health care cases of ASD in the Stockholm population may go undetected, especially among children with ASD without ID, which may lead to misclassification: parents with a child with an undetected ASD classified as not having a child with ASD will result in weakened associations. Additionally, selection bias might occur since we exclude 11,586 mother/fathers because one or both of them have children with another partner, keeping only the “first” family, in order to prevent parents from being counted more than once. Since the likelihood of having a child with ASD increases with age (Croen et al. 2007), and since these parents are likely to be older, we may have selected a larger number of parents who did not have a child with ASD than if we had instead chosen to keep the most recent family. However, there is no reason to believe that this group differs regarding the outcomes.

To make the comparison group representative of the Stockholm population we included parents with children with other disabilities. Some parents in the comparison group may have had children with other conditions which could have affected the four outcomes, possibly attenuating the results. It would have been interesting to compare parents who have children with other (non ASD) disabilities as well as to study comorbidities among the children with ASD (for example OCD, anxiety, and depression.). However, data for these other diagnoses were not readily available, but could perhaps be studied in the future.

Another limitation arises from the complicated nature of the outcomes. We do not know why people are sick or why they are not in the labor force. Not being employed will affect well-being in different ways depending on whether or not the person is willingly or unwillingly outside of the labor force. Also, while having an income below the 20th percentile very likely indicates part time work, we do not know how many hours parents work or if they work more hours but at a very low wage. Additionally, parents in well paid jobs may work part time but exceed the 20 %, thus for these reasons low income is a crude measure for work participation.

We controlled for a variety of possible confounders such as being born outside of Sweden, socioeconomic factors (level of education, single parent household, receiving social assistance), and parental psychiatric contact. However, there are many other factors which can contribute and are not covered in the scope of this study that might be better suited for a qualitative study.

### Conclusions

In conclusion, this study found that being a parent of a child with ASD is associated with higher maternal and paternal sick leave and lower work participation. The study found that ASD without ID but not ASD with ID was associated with sick leave lasting 15–365 days, but that both ASD with and without ID were associated with long term sick leave, not being in the labor force and low income. The findings are of particular interest because they appear in a society that has developed many policies to support parents with children with disabilities. In such a society it might be expected that these parents have comparable levels of sick leave, being in the work force, and income. However our findings suggest that despite Swedish policies aimed at helping families of children with ASD, both with well-being and with ability to work, that these parents remain a vulnerable group for which additional support might be warranted. It can also be noted that being on sick leave, outside of the work force or earning a low income will have long reaching impact on these parents because of Sweden’s pension system which is based on an individual’s life time earnings. It is recommended that further studies be done to see what support mothers and fathers would find most beneficial and what support they are lacking.
